# Transitioning from the “Three Delays” to a focus on continuity of care: a qualitative analysis of maternal deaths in rural Pakistan and Mozambique

**DOI:** 10.1186/s12884-023-06055-w

**Published:** 2023-10-23

**Authors:** Marianne Vidler, Mai-Lei Woo Kinshella, Esperanca Sevene, Gwyneth Lewis, Peter von Dadelszen, Zulfiqar Bhutta, the CLIP Working Group, Esperança Sevene, Eusébio Macete, Khátia Munguambe, Charfudin Sacoor, Anifa Vala, Helena Boene, Felizarda Amose, Rosa Pires, Zefanias Nhamirre, Marta Macamo, Rogério Chiaú, Analisa Matavele, Faustino Vilanculo, Ariel Nhancolo, Silvestre Cutana, Ernesto Mandlate, Salésio Macuacua, Cassimo Bique, Sibone Mocumbi, Emília Gonçálves, Sónia Maculuve, Ana Ilda Biz, Dulce Mulungo, Orvalho Augusto, Paulo Filimone, Vivalde Nobela, Corsino Tchavana, Cláudio Nkumbula, Rahat Qureshi, Zulfiqar A. Bhutta, Zahra Hoodbhoy, Farrukh Raza, Sana Sheikh, Javed Memon, Imran Ahmed, Amjad Hussain, Mrutunjaya B. Bellad, Umesh S. Charantimath, Shivaprasad S. Goudar, Geetanjali M. Katageri, Avinash J. Kavi, Amit P. Revankar, Ashalata A. Mallapur, Umesh Y. Ramdurg, Shashidhar G. Bannale, Vaibhav B. Dhamanekar, Geetanjali I. Mungarwadi, Narayan V. Honnungar, Bhalachandra S. Kodkany, Anjali M. Joshi, Uday S. Kudachi, Sphoorthi S. Mastiholi, Chandrappa C. Karadiguddi, Gudadayya S. Kengapur, Namdev A. Kamble, Keval S. Chougala, Jeffrey Bone, Dustin T. Dunsmuir, Sharla K. Drebit, Chirag Kariya, Tang Lee, Jing Li, Mansun Lui, Beth A. Payne, Diane Sawchuck, Sumedha Sharma, Domena K. Tu, Ugochi V·Ukah, Laura A. Magee, JMark Ansermino, Ana Pilar Betrán, Richard Derman, Shafik Dharamsi, France Donnay, Sharla Drebit, Guy Dumont, Susheela M. Engelbrecht, Veronique Fillipi, Tabassum Firoz, William Grobman, Marian Knight, Ana Langer, Simon Lewin, Craig Mitton, Nadine Schuurman, Andrew Shennan, Joel Singer, Jim Thornton, Hubert Wong, Olalekan O. Adetoro, Khátia Munguambe, John O. Sotunsa

**Affiliations:** 1 Department of Obstetrics and Gynecology, University of British Columbia, Vancouver, Canada; 2 Faculty of Medicine, Eduardo Mondlane University, Maputo, Mozambique; 3 Centro de Investigação Em Saúde da Manhiça, Manhiça, Mozambique; 4 Bournemouth University, Poole, UK; 5 Department of Women & Children’s Health, King’s College London, London, UK; 6 Department of Pediatrics, Aga Khan University, Karachi, Pakistan; 7 Centre for Global Child Health, Hospital for Sick Children, Toronto, Canada

**Keywords:** Maternal mortality, Quality of health care, Continuity of patient care, Narrative analysis, Maternal health, Cause of death, Postnatal period, Developing countries

## Abstract

**Background:**

The Three Delays Framework was instrumental in the reduction of maternal mortality leading up to, and during the Millennium Development Goals. However, this paper suggests the original framework might be reconsidered, now that most mothers give birth in facilities, the quality and continuity of the clinical care is of growing importance.

**Methods:**

The paper explores the factors that contributed to maternal deaths in rural Pakistan and Mozambique, using 76 verbal autopsy narratives from the Community Level Interventions for Pre-eclampsia (CLIP) Trial.

**Results:**

Qualitative analysis of these maternal death narratives in both countries reveals an interplay of various influences, such as, underlying risks and comorbidities, temporary improvements after seeking care, gaps in quality care in emergencies, convoluted referral systems, and arrival at the final facility in critical condition. Evaluation of these narratives helps to reframe the pathways of maternal mortality beyond a single journey of care-seeking, to update the categories of seeking, reaching and receiving care.

**Conclusions:**

There is a need to supplement the pioneering “Three Delays Framework” to include focusing on continuity of care and the “Four Critical Connection Points”: (1) between the stages of pregnancy, (2) between families and health care workers, (3) between health care facilities and (4) between multiple care-seeking journeys.

**Trial registration:**

NCT01911494, Date Registered 30/07/2013.

## Background

Husna developed labour pains and vaginal bleeding in her ninth month of pregnancy in Sindh Province, Pakistan. Her mother-in-law recalled that they took her to three private hospitals in search for care, but none could perform an ultrasound and would not deliver her due to concerns about umbilical cord prolapse. Husna’s husband, the decision-maker for the household, suggested going to the government tertiary hospital where doctors performed an emergency Caesarean. Husna’s recovery was reportedly complicated by low blood pressure. The “*doctors were showing carelessness and were reluctant*,” Husna’s mother-in-law said, and “*Doctors themselves seemed to be worried. We still requested them to check her*.” When the doctor finally came to check on her, Husna was already dead.

Husna’s narrative demonstrates some of the challenges in accessing quality maternal care in rural Pakistan. This excerpt reveals the frenetic journey from the onset of the obstetric complication to its tragic outcome. With maternal mortality ratio (MMR) estimates of 349 deaths per 100,000 livebirths, Pakistan has one of the highest MMRs in the world [1]. Rural Pakistan is largely underserved with an MMR almost twice that of urban areas [2]. Pregnant and postpartum individuals face similar challenges in Mozambique where there is an MMR of 327 per 100,000 livebirths. The highest rates of maternal mortality have been found in southern regions of Mozambique [3].

This paper explores the interpersonal and systemic factors that contributed to maternal deaths in rural Pakistan and Mozambique, during the Community Level Interventions for Pre-eclampsia (CLIP) Trial (NCT01911494), through analysis of verbal autopsy narratives. While the Three Delays Framework galvanized momentum to reduce global maternal mortality leading up to and during the Millennium Development Goals (MDGs), the verbal autopsy narratives of 76 maternal deaths in rural Pakistan and Mozambique expose a need to modernize the framework to highlight the importance of continuity of care and the complexity of care pathways. We will provide a brief history of the Three Delays Framework and then present the narrative analysis of maternal deaths in the CLIP Trial and recommend updates to the framework two decades after Thaddeus and Maine’s landmark publication [4].

### Development of the Three Delays Framework

At the 1974 National Conference for International Health, then Director-General of the World Health Organization (WHO), Halfdan T. Mahler described how the Western medical model with medical centres concentrated in urban areas, physician-led treatments and modern technologies as promoted in the 1950s and ‘60 s was not suited to less-developed settings. This approach created “disease palaces” that consumed the health budgets of poor nations yet ignored the needs of most people who were predominantly rural in residence. A solution to this gap in rural care was a shift in focus to primary health care in rural settings; the Alma Ata conference in 1978 adopted the approach as a global strategy. However, in 1985, Rosenfield and Maine observed that the increase in maternal and child health programs had not reduced maternal mortality because primary health centres focused on preventative care and management of childhood infections and malnutrition [5]. Rosenfield and Maine called maternal mortality a “neglected tragedy,” and issued a call for major investment in comprehensive maternity care [5].

This call to action was taken up by Mahmoud Fathalla, the Director of the WHO’s Special Programme of Research Development and Research Training in Human Reproduction, who helped to launch the Safe Motherhood Initiative (SMI) in 1987 [6]. Building advocacy around maternal mortality since Rosenfield and Maine’s 1985 article [7], the SMI was the first to focus on the prevention of maternal deaths, “not because death adversely affects children and other family members, but because women are intrinsically valuable” [4]. Fathalla’s “road to death” recognized that poor socio-economic status compounded by high parity can increase the likelihood of a high-risk pregnancy and maternal death [8].

Building off Fathalla’s conceptualization of a pathway of factors that influence maternal mortality [8] and the first five years of the SMI, McCarthy and Maine developed a “first generation” maternal mortality determinants framework in 1992 [9]. Their framework concluded that all efforts to reduce maternal mortality work through reducing the likelihood that someone will become pregnant, reducing the likelihood that a pregnant individual will experience a serious complication and improving outcomes for those with complications. Thaddeus and Maine further focused on improving the management of obstetric complications as the key to reducing maternal deaths [4]. At the time, the vast majority of maternal deaths resulted from direct obstetric causes (hemorrhage, obstructed labour, infection, pre-eclampsia/eclampsia, unsafe abortion) and could have been prevented with timely treatment. Thaddeus and Maine outlined three critical delays:

1. The first delay is deciding to seek care, which includes a) socio-cultural factors such as recognition of complications and perceived severity of illness, the actors involved in decision-making and the status of women, b) perceived accessibility around distance, transport and the associated costs, and c) perceived quality of care.
2. The second delay is in identifying and reaching a health facility, which includes issues around a) distribution and location of facilities, b) distance and travel time, c) availability of transport and road conditions, and d) costs of transport.
3. The third delay is receiving adequate care at the facility, which includes issues around a) poorly staffed facilities and competence of personnel, b) shortages of supplies and equipment, and c) inadequate management.

The Three Delays Framework was revolutionary. Use of the framework has increased significantly since 2004 [7], which coincided with the MDGs. From a “neglected tragedy” [5], reducing maternal mortality became prioritized as one of the MDGs, which was a monumental achievement of SMI. Development of the verbal autopsy (VA) was in part to track progress of the MDGs, including maternal mortality [10].

### Updates and alternatives to the Three Delays Framework

Like the original Three Delays Framework, Gabrysch and Campbell explored factors associated with reaching health facilities [11]. The Three Delays Framework assumed that people were giving birth at home, thus the first and second delays of getting to a health facility [11]. However, if women were already at the facility for delivery, the key issue becomes the ability to provide appropriate care. While time may be critical in emergencies, perceptions and physical access were greater concerns in preventative care-seeking [11]. Perceptions included perceived quality of care, and perceived benefits and needs weighed against opportunity and transport costs.

A 2012 review of 58 maternal verbal autopsies (VA) in Malawi recognized that the Three Delays Framework did not sufficiently address accessing antenatal or postnatal care [12]. Additionally, they found that indirect causes of maternal deaths, such as anemia, HIV/AIDS and chronic infections were not adequately captured [12].

Pacagnella and colleagues critiqued the Three Delays Framework for not accounting for primary prevention or early disease detection [7]. They added a fourth delay to explore the consequences of maternal morbidities, which may include the economic burden of care, underlying diseases or consequences of the lifesaving interventions. Building on Pacagnella et al. [7], MacDonald et al. expanded the fourth delay to include the community’s role in preventing maternal deaths and collective engagement to mobilize resources [13].

In contrast to increased focus on community factors, Knight, Self and Kennedy reflected that the Three Delays Framework has focused on care-seeking, which they termed as ‘supply-side’, while quality of care, which they termed as ‘demand-side’, was neglected [14]. Their review of 43 articles on the third delay found inadequate training, drug procurement and logistic problems, staff shortages, lack of equipment, and low staff motivation as the top five facility-level barriers [14].

Ely Yamin reconceptualised the Three Delays Framework in terms of a lack of available, accessible, acceptable and quality health care instead of individual behavioural factors [15]. She pointed out how the low status of women influences the decision to seek care as they rarely had the power to make the decision and low political priority to make maternal healthcare adequate [15].

Bhutta and colleagues expanded on the Three Delays Framework to highlight the difference between disease recognition and decision-making, which had been traditionally grouped together as the first delay [16]. When the first delay was broken into these two components, Bhutta and colleagues (2013) found that 18% of maternal deaths in Pakistan experienced a delay in recognition and 34% experienced a delay in decision-making. Many women reported delays due to availability or cost of transport (80%) and accessing appropriate and prompt health services (73%). These findings highlight the need to strengthen health systems and improve quality of care. These results also illustrate the multiple delays women face, this reinforces Fathalla’s (1987) maternal death road and how multiple factors may compound risk.

The evolution of the Three Delays Framework demonstrates its sustained influence on the field of maternal health. The Three Delays Framework, as proposed by Thaddeus and Maine [4], contains four implicit assumptions that 1) women are giving birth at home [11], 2) all pregnant woman are at equal risk of death [17], 3) deaths are due to direct causes [4], and 4) time is the key determinant [7]. These assumptions signify a gap in incorporating trends and developments in maternal health in the last two decades. This paper will review a subset of maternal deaths in rural Pakistan and Mozambique to understand the context of their tragic outcomes with attention to these assumptions.

## Methods

Verbal autopsies of maternal deaths were performed during the CLIP Trial in Pakistan and Mozambique (2015–17, NCT01911494). The CLIP cluster randomized controlled trial evaluated a package of community engagement and mobile health guided clinical management to reduce maternal and perinatal death and morbidity among 54,459 pregnancies enrolled (39,446 in Pakistan and 15,013 in Mozambique), the trial is reported in detail elsewhere [18–20]. Informed consent to participate was obtained from all study participants.

The study was conducted in Hyderabad and Matiari in Sindh Province, Pakistan and Maputo and Gaza Provinces in Mozambique. Hyderabad and Matiari are southern districts in Sindh, with Hyderabad district including the city of Hyderabad and the farmlands to the southeast. Matiari is a rural district located 25 kms north of Hyderabad [21]. People living in these districts are predominantly Muslim and work largely in the agricultural sector [21]. The health system is a mix of private and public facilities and services [22]. Maputo and Gaza Provinces are rural regions in southern Mozambique. Most individuals work in the agriculture sector, and men regularly travel outside the country for employment [23]. The health system is made up of predominantly public facilities with some private hospitals in urban centers. During the rainy season, November-March, some regions experience extreme rain and flooding which can make major transport routes impassible [24].

The 2012 WHO verbal autopsy was administered in cases of maternal death in the CLIP Trial, this included the open-ended question: “Could you tell me about the illness/events that led to her death?” [10]. Data collectors interviewed husbands, mothers, or other family members of the deceased individual. Narrative responses were transcribed, translated in to English and analyzed using NVivo 12 software (QSR International, Melbourne, Australia) with a coding framework developed through an iterative process. Preliminary analyses were coded according to the Three Delays Framework; however, the narratives did not neatly map onto delays of deciding to seek care, reaching care and adequate treatment. The first ten narratives were reviewed independently by two qualitative researchers (MWK, MV). When interrater reliability was confirmed at ten narratives, one researcher (MWK) completed coding for the remaining cases while the second researcher (MV) coded a random selection of narratives to ensure continued interrater reliability. A second round of analysis was completed to explore the themes and details that emerged. All names are pseudonyms to protect confidentiality.

## Results

There were 106 maternal deaths across the 20 clusters in the CLIP Pakistan Trial [MMR of 305.5 per 100,000 live births] and 22 maternal deaths across 12 clusters in the CLIP Mozambique Trial (MMR of 174 per 100,000 live births). Fifty-four VAs (51%) included narratives in Pakistan and all VAs in Mozambique included narratives. In Pakistan, VA narratives were not collected in the pilot phase of the trial and six clusters did not collect VA narratives.

The median age of maternal deaths was 30 years in Pakistan and 27 in Mozambique, compared with 28 and 23 years in the overall trial cohort respectively. Most women who died in Pakistan had no schooling (83%), similar to the overall trial cohort (82%), while 55% of the women who died in Mozambique had completed primary education, which was fewer than the trial cohort (68%) [18, 20]. Most deaths were postpartum (69% in Pakistan and 73% in Mozambique), usually within the first 24 h of delivery and usually at facility; very few deaths occurred at home (11/76, 14%) (Table 1).

**Table 1:** Maternal characteristics^a^

		**Pakistan** (*N* = 54)		**Mozambique** (*N* = 22)		**Total** (*N* = 76)	
		n	%	n	%	n	%
**Maternal age**	Median [IQR]	30 years [25, 32]		27 years [23,34]		30 years [25,33]	
	≥ 35 yrs	11	20%	5	23%	16	21%
**Maternal education**	No schooling (0 yrs)	45	83%	7	32%	52	68%
	Completed primary education (≥ 5 yrs)	6	11%	12	55%	18	24%
	Other	3	6%	3	14%	6	8%
**Parity**	First pregnancy	8	19%	6	27%	14	18%
	2–3	5	12%	4	18%	9	12%
	4–5	13	31%	7	32%	20	26%
	≥ 6	16	38%	5	23%	21	28%
	Missing	12^b^	-	0	-	12	16%
**Timing of death**	Antepartum	11	20%	1	5%	12	16%
	Intrapartum	6	11%	4	18%	10	13%
	Postpartum	37	69%	16	73%	53	70%
	Unknown	-	-	1	5%	1	1%
**Timing of postpartum deaths**	First 24 h	23	42%	6^c^	38%	29	38%
	25—48 h	1	2%	1	6%	2	3%
	2—7 days	2	4%	2	13%	4	5%
	8—42 days	10	19%	6	38%	16	21%
	Unknown	-	-	1	6%	1	1%
**Location of death**	At home before seeking care	2	4%	5^d^	23%	11	14%
	At home post-discharge	4	7%				
	En route	11	20%	1	5%	12	16%
	At facility	37	69%	16	73%	53	70%

^a^Data obtained from narratives as shared by family members of the woman who died in Pakistan and from household surveillance data in Mozambique

^b^Described in 42 of 54 narratives, 78%

^c^All same day, uncertain if they were postpartum or intrapartum

^d^Timing is unknown, may or may not be after a facility discharge

### Critical connection point 1: between stages of pregnancy

The maternal death narratives revealed that many women in both settings suffered from underlying conditions or risk factors that may not have been well followed in pregnancy. Nearly half (46%) of the cases were first time pregnancies (18%) or sixth or greater (28%). Over a quarter of narratives in Pakistan (15/54, 28%) discussed a previous miscarriage or stillbirth. Comorbidities were commonly reported and included anaemia, diabetes (“high sugar”), hepatitis, high and low blood pressure, HIV, jaundice or kidney disease, malaria, asthma and whooping cough. Both high and low blood pressure were discussed most frequently (7/54, 13% of cases each), followed by anaemia (6/54, 11%) in Pakistan. Malaria (3/22, 14%) and HIV (6/22, 28%) were the most common comorbidities discussed in Mozambique.

For example, in Mozambique Elisa’s mother-in-law shared that, “*During the pregnancy, she complained about headaches, recent malaria, and later she went to the hospital for further investigation and it was found that she was HIV positive and could start the treatment, but she refused it and went to South Africa where she was staying at the time. The disease got worse and her relatives asked her to come back to Mozambique. [We] took her to the hospital, where she had the treatment, and went back home. One month after she gave birth, the disease worsened even more and she lost her life…*” In some cases, the diagnosis was not disclosed during pregnancy which may have complicated care, such as with Sara in Mozambique: “*It is suspected that she had HIV, but she was not being treated. After the funeral, they found her health card and pills under her bed”*.

Returning to Husna’s narrative in Pakistan, it was reportedly her fourth pregnancy, with two living daughters and one previous miscarriage. “*In the beginning of this pregnancy*,” her mother-in-law recalled, “*she had headache, pain in neck, darkness in front of eyes, backache and pain in lower abdomen…Doctor used to check her and say her BP (blood pressure) was low*.” The doctor also said she had anaemia and slight jaundice, the latter of which the doctor said they would treat after pregnancy.

Family members often recounted warning symptoms; there were only five narratives (four in Pakistan and one in Mozambique) in which families reported that neither the doctor nor the patient reported any problems. There were only two cases where the woman appeared to have no problems throughout pregnancy or delivery, and the maternal death was perceived to come with no warning. 

### Critical connection point 2: between family members and health care providers

In many instances patients and families were not well informed of the conditions leading to death or did not comprehend the information provided, highlighting a gap in communication between health care providers and families. For example, in Husna’s narrative, her mother-in-law recounted that medication was prescribed to normalize her blood pressure, but she did not appear to fully understand the clinical diagnosis. In the report of Husna’s symptoms, her surviving family members did not to differentiate what could have been normal pregnancy symptoms, such as constipation, from symptoms that may have been indicative of pre-eclampsia, such as continuous headache and visual disturbances, epigastric pain, and swelling in her hands and feet.

In Mozambique, one father described that “*the family did not know what disease led to her death”*. Additionally, the tragic story of Graça’s death in Mozambique highlights the disconnect and challenges in communication between patients, family members and health care providers: *“She didn’t go back home and was transferred to the Provincial Hospital […], and from that day on, none of her relatives heard about her since she didn’t have a cellphone to be contacted, and worse than that, she was all alone during this process. Later on, it was communicated on the radio that she lost her life”*.

### Critical connection point 3: between health facilities

A lack of health system capacity and skills to manage complications resulted in several out-referrals, often accompanied with poor communication. Twenty narratives (20/54, 37%) in Pakistan described a lack of skill or capacity during emergencies. In Pakistan, six narratives (6/54, 11%) described a facility’s refusal to treat patients when the condition was severe, and many were referred between facilities when the situation became too serious for the first facility to handle (29/54, 54%). At times, there were also delays attributed to clinical decision-making at the facility: the doctor thought the woman was well or did not realize the severity of the condition. A majority of visits in Pakistan were to private health facilities (88/146, 60%) with 58 visits (40%) to a publicly-funded facility. In Husna’s case, even though she lived within 30 kms of Hyderabad city, she visited four different health facilities, three of which were private. The multitude of private facilities with differing levels of services and the high rates of referral led to convoluted and chaotic pathways to care.

Husna’s narrative, spoke of a chaotic journey in search for care. Her mother-in-law recalled how the doctor at the first private clinic stated: “*Kindly get her an ultrasound because without an ultrasound, we will be unable to perform her delivery, as well as due to vaginal bleeding, we will be unable to do her delivery*” indicating a lack of capacity to manage obstetric complications. The second private hospital was unable to do an ultrasound and ultrasounds were not available at night in the third private hospital. She had lost a lot of blood by the time she reached the fourth facility, and although the need to get an ultrasound was a major reason for being shuffled between health facilities, there was no indication she received an ultrasound once finally admitted.

Similarly, the majority of maternal deaths in Mozambique (18/22, 82%) included at least one referral. Many of these referrals were to higher levels of care for caesarean section or other unspecified operation (6/22, 27%). The case of Andreia illustrates the complicated health system patients and families must navigate. Andreia’s mother described a wound she suffered in pregnancy, she “*went to the local health unit, was seen by a doctor and went back home. She got a little bit better and kept her normal routine but the wound didn’t heal*”. Subsequently, Andreia sought care again at her local hospital, where she was transferred, treated and discharged home. “*Without any signs of improvement, [she] went back to the local health unit”* where she was referred two more times and ultimately lost her life.

In some cases, the referral pathways changed in fundamental ways from day-to-day. Referrals also frequently required additional transport. Although few narratives (15/76, 20%) mentioned the means of transport, including ambulance, motorbike, rickshaw, vehicle and walking, the most common was hiring a vehicle (7/15, 47%), which was sometimes delayed. Ambulance services (3/15, 20%) were only available between government hospitals. Facilities were less likely to be open at night or during holidays. Traffic was mentioned only once. A substantial proportion of deaths occurred en route to care or during referral between facilities (12/54, 16%). The high referral rates and convoluted pathways exacerbated delays, leading many to arrive at the final facility in critical condition.

### Critical connection point 4: between care-seeking journeys

Maternal deaths were rarely the result of a single care seeking event. Family members described cycles of health problems, visiting a doctor, receiving treatment, temporary improvement in some cases, and re-emergence or worsening of the condition. There was frequently no connection or communication between health care seeking journeys complicating care.

Many women shared their health concern with their physician and received treatment that improved their condition temporarily. In Pakistan, many of these cases were related to blood pressure treatment. For example, early in Husna’s pregnancy, “*Doctor prescribed medicines to normalize her BP and for stomach problems. By taking those medicines, she was temporally relieved from her symptoms*.” In her last trimester, “*Doctor used to prescribe medicines, and on taking those, she was relieved for some time*.” Sixty-nine percent (37/54) of narratives in Pakistan included at least one instance of temporary improvement after treatment, with two or three recurrences in some narratives. Seven percent of maternal deaths in Pakistan (4/54 including two postpartum) occurred at home after discharge or after the doctor indicated the patient was fine. In some cases, temporary improvements and ultrasounds provided a false sense of security; mentioning that women “felt fine” or did not think complications were serious were the most common reasons to delay care-seeking (25/54). Ultrasounds were discussed in 50 of 54 narratives in Pakistan. Fourteen percent (7/50) reported five or more ultrasounds and family members often stated that the doctor recounted the baby was fine. Husna’s mother-in-law reported three antenatal care (ANC) visits, getting vaccinations and two ultrasounds during this pregnancy. After completion of nine months gestation, Husna developed low intensity labour pains. The family waited until the third day when they became more frequent and accompanied by vaginal bleeding before seeking care.

Thirty-two percent of cases (7/22) included multiple care journeys in Mozambique; however, they less often included improvements in symptoms after discharge, such as with Zaida: *“She bought a few pills and medicated herself, but the disease wouldn´t cease”*.

### Rehema’s story

Rehema’s narrative brings the themes together, which the paper has illustrated through Husna’s story in Pakistan and the stories of Elisa, Sara, Graça, Andreia and Zaida in Mozambique. Rehema’s story demonstrates how the factors interact and compound risk. Rehema was 35-years-old with no formal schooling on her twelfth pregnancy. Seven of her children were alive; she had had three previous miscarriages and one stillbirth.

“*During early months of this pregnancy, after five months Rehema had pain in her legs and felt weak and sweat with little excursion. For check-ups, she went to the government hospital. Doctor checked her and said her BP (blood pressure) was low and prescribed medication. She took those pills and was feeling well temporarily. During the last trimester of this pregnancy, Rehema had pain in her limbs, weakness, profuse sweating with minimal movement and her body became very cold. We gave her Tang (glucose with Vitamin C) and she was relieved for some time... She visited the government hospital. Doctor checked her and said her BP is low and prescribed pills, which she was taking and her symptoms subsided for some time. During this pregnancy, Rehema had an ultrasound three times; doctor said baby is well... Rehema also had vaccinations. When she completed nine months, Rehema had labour pains in morning at 11 am…[and] had foul smelling vaginal discharge. We gave her castor oil as an enema so that she could deliver normally, then took her to a private clinic where the doctor admitted her…One injection and one drip was given, then the doctor delivered her vaginally. Baby cried soon after the delivery and was taking breaths normally…baby is alive!... After some time, Rehema had anxiety and was shouting. She was sweating profusely, was absolutely cold and crying. We asked the doctor to check her…The doctor then reported that “Rehema has very low BP and this case is beyond my control so please take her to some other health facility”. Then, we took her to another private hospital. When we reached the gate of the hospital, Rehema expired. We still requested the doctor to see her. The doctor checked her and said, “You have brought her dead…We are from God and we shall return back to Him!*”

Rehema had underlying risk factors, including advanced maternal age, high parity and a history of miscarriages and stillbirth. She had symptoms in early and late pregnancy, for which she sought medical care and received treatment that led to temporary improvement. However, she may not have sought care until after five months when symptoms first arose and undertook alternative therapies, including Tang beverage and castor oil enema. Her family highlighted three ultrasounds, where the doctor confirmed the baby was fine after each. Although she attended antenatal care at government hospitals, she went to a private hospital to deliver. When complications arose after delivery, the doctor requested that Rehema be taken elsewhere; tragically, she died shortly before arrival.

## Discussion

**Fig. 1:**
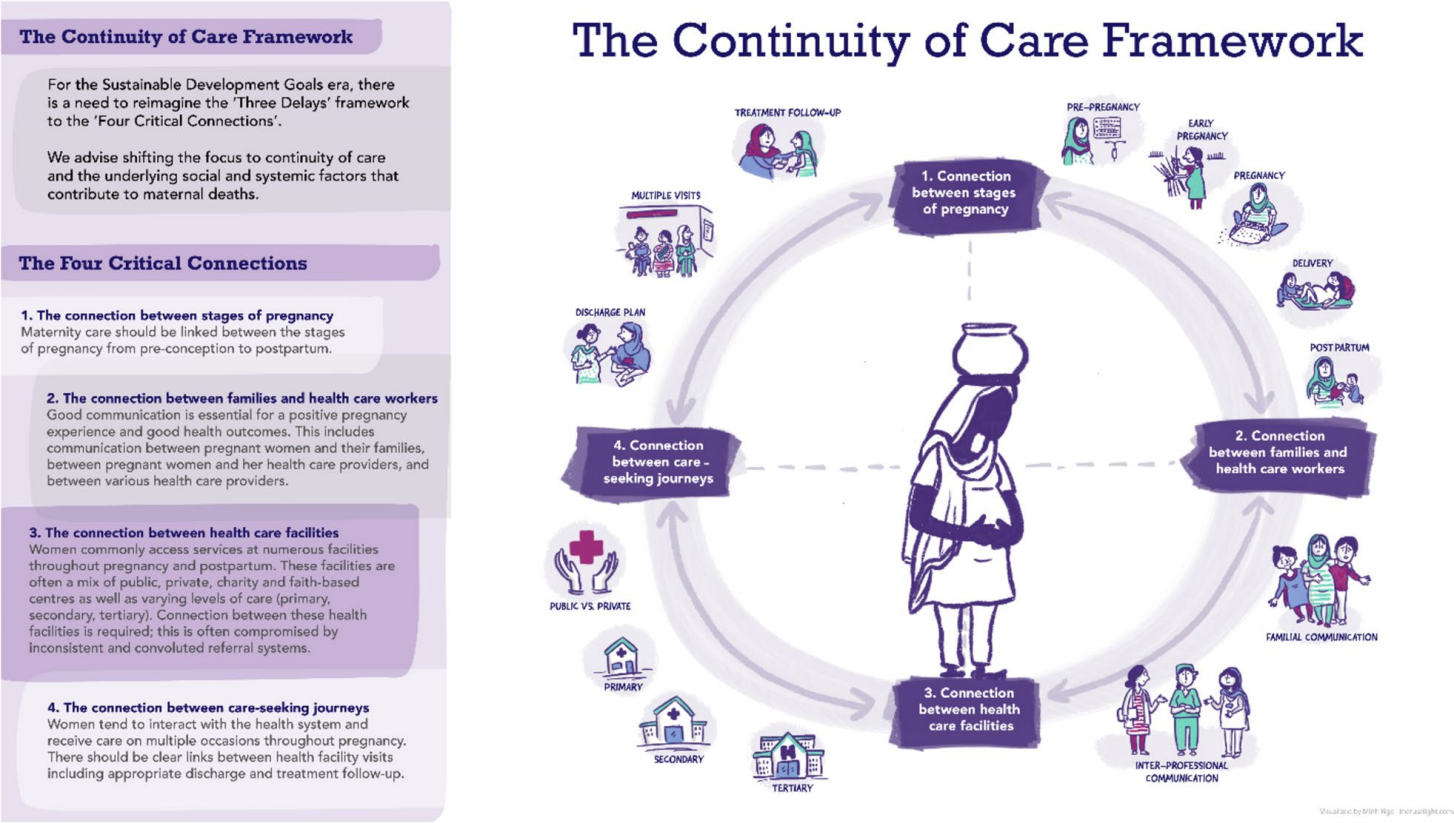
The Continuity of Care Framework

The stories presented here highlight the importance of underlying risk factors, access to care, and the quality and continuity of clinical services in rural Pakistan and Mozambique. Thaddeus and Maine likewise found that women arrived at the final facility in critical conditions [4]. However, the narratives presented here revealed that women typically had many interactions with the health system before death, instances of temporary improvement, discharge and referral through convoluted systems, the lack of continuity in their was critical in their journeys. Most deaths were postpartum though complications often originated antepartum, and most of these women appeared to have underlying comorbidities. Evaluation of these narratives helps to reimagine the pathways of maternal mortality beyond a single care-seeking journey, to expand the categories of seeking, reaching and receiving care to a focus on continuity of care and critical connection points in clinical service (Fig. 1).

This analysis of VA narratives challenges the assumptions of the Three Delays Framework. First is the assumption of homebirths. When the Three Delays Framework was first published in 1994, there were high rates of home births but with targeted efforts to promote facility birth as part of the MDGs [15], rates of institutional delivery dramatically increased. Only three home births were recorded among the maternal deaths in this study, giving an institutional delivery rate amongst the women who died of 93%. Two other qualitative analyses of VAs likewise found that women sought care in emergencies but faced barriers in access and quality of services [25, 26].

The frequent mentions of comorbidities, the high rate of first pregnancies or high parity and history of miscarriage or stillbirth suggest that women were not at equal baseline risk and consequently, early detection, management and birth preparedness have a potential role. While this study did not look at the causes of death, narratives often described pre-existing conditions, such as anaemia and hepatitis. The Three Delays Framework is built on the foundation that a vast majority of maternal deaths are from direct causes [4], warranting a focus on emergency obstetric complications [17]. Over 70% of maternal deaths are from direct causes; however, the 27.5% from indirect causes are of growing importance, and need addressing, especially as health services start to manage the major direct causes of death [27]. The proportion of indirect causes will increase as targeted interventions decrease direct maternal deaths. Seventy percent of indirect maternal deaths are due to pre-existing disorders, such as HIV, exacerbated by pregnancy [27]. The Three Delays Framework was published during the beginning of the HIV/AIDS epidemic, which reshaped the field of global health and heightened awareness of health system failures and social inequities [28].

In contrast to conceptualizing a single journey of care-seeking, narratives in this study revealed a number of disconnected loops and cycles. This fits into the larger critique that the Three Delays Framework focused most on emergency obstetric care and did not adequately address issues related to antenatal and postnatal care [7, 12]. The narratives in this study revealed that treatment and temporary improvement without comprehensive antenatal care may contribute to a false sense of comfort and delay care-seeking. Many women experienced symptoms and complications, often long before their death. In contrast to typical conceptualizations that a lack of awareness about maternal health issues led to delays in seeking care, their many interactions with the medical system may have contributed to the sense that conditions were normal. A second potential loop in the care journey consisted of inappropriate discharges and clinicians’ perceptions that the condition was not serious, which may lead to further complications at home and re-initiation of the care-seeking journey. Third, there were loops within the convoluted referral system as patients are passed between facilities in critical condition. The challenges in accessing care often focused on getting to the *right* health facility. The narratives demonstrate what others have described, that the poor referral systems between communities, rural health services and secondary hospitals impede effective maternal and newborn care [16]. Lastly, the narratives indicate that the journeys of care do not end with delivery, there were several late maternal deaths, which involved additional care cycles after delivery.

These loops and referrals, that women must navigate, reveal serious issues regarding continuity of care. The woman and her family carry the burden to provide important medical information to the next clinician, a process hampered by limited education, medical literacy, and social class inequities. While testimonies of poor quality of care at facilities may highlight problems with clinicians, Yamin [15] argues that accountability must move beyond simply “naming and shaming” to understand the systemic failures that health care professionals also work with. It is important to recognize that clinicians may not be privy to the entire clinical picture and trajectory before reaching the facility, which compromises their capacity for effective care. Secondly, the health landscape featured a confusing array of private and public health facilities. In Pakistan, a majority of visits were to private facilities; however, most narratives involved visits to both private and public facilities. Pakistan’s private sector grew to accommodate health system gaps as population growth outpaced government investment and private facilities are perceived to have better quality of care, but the private sector is unregulated and many have questionable quality [29, 30].

The narratives found that doctors referred out or refused service when they felt that the situation was beyond their ability to provide care. The practice of referring out difficult cases was evident in all levels of the health system. Increasingly, health metrics and indicators are used for assessments of quality and performance. Subsequently, MMRs can become politicized [31] and tied into funding [32]. A medical anthropologist in Nigeria documented how a maternal death remained unrecorded to avoid adding another maternal death to the facility’s statistics [32]. Health statistics, consequently, “not only have the power to determine which interventions are successes, which are failures, which will be funded and which will not, but they also carry the political clout to determine who will get re-elected to office, who will be promoted to chief medical director of a hospital, and who will win a government contract” [32]. For some clinicians in this study, there may have been a desire not to have an additional maternal death on their facility’s statistics.

While the narratives discussed critical events in the health journey, the timing of these events was not always known. Additionally, these were clinical narratives and socio-economic and cultural factors were not well explored. The narratives were told by the deceased woman’s next of kin, often mothers-in-law and husbands, and share their perspectives, which may highlight different factors [33]. Self-reporting of symptoms and medical history may be influenced by the education level, social desirability and time since the event.

## Conclusion

Though MDG 5, to reduce MMR by three quarters, was ultimately not achieved, global MMR significantly declined by 44%, from 385 to 216 deaths per 100,000 live births [27]. The substantive MMR reduction represents the success of the Three Delays Framework, which has been influential in framing determinants of maternal mortality, and targeting efforts to meet MDG 5. Dramatic increases in institutional deliveries, antenatal care attendance and findings from this and other studies suggest that women are regularly accessing care. However, increasing care-seeking without improving health systems can lead to gaps in the quality of that care.

Seventy-six maternal death narratives demonstrate interconnected challenges that weave throughout the community and facilities in loops and cycles rather than a unidirectional journey. The stories presented here stand witness to their tragic journeys in hopes of preventing similar deaths in the future. Reimagining the Three Delays Framework should shift away from individual-level factors and the silos of seeking, reaching and receiving care to a focus on the health system and the numerous multi-directional events of care-seeking. We propose an updated framework that focuses on continuity of care and four key connection points: between stages of pregnancy, between families and health care workers, between health facilities, and between care seeking journeys. This framework can be used to understand and reduce maternal deaths in the context of clinical services in low- and middle-income countries, such as Pakistan and Mozambique.

## Data Availability

The datasets used and analysed during the current study are available from the corresponding author on reasonable request.

## Abbreviations

MMR: Maternal mortality ratio
CLIP: Community Level Interventions for Pre-eclampsia
MDG: Millennium Development Goals
WHO: World Health Organization
SMI: Safe Motherhood Initiative
VA: Verbal autopsy
ANC: Antenatal care
BP: Blood pressure
